# Enolase 1 regulates stem cell-like properties in gastric cancer cells by stimulating glycolysis

**DOI:** 10.1038/s41419-020-03087-4

**Published:** 2020-10-16

**Authors:** Ting Yang, Xiong Shu, Hui-Wen Zhang, Li-Xin Sun, Long Yu, Jun Liu, Li-Chao Sun, Zhi-Hua Yang, Yu-Liang Ran

**Affiliations:** 1 State Key Laboratory of Molecular Oncology, National Cancer Center/National Clinical Research Center for Cancer/Cancer Hospital, Chinese Academy of Medical Sciences and Peking Union Medical College, Beijing, 100021 China; 2 Laboratory of Molecular orthopaedics, Beijing Research Institute of Orthopaedics and Traumatology, Beijing Ji Shui Tan Hospital, Beijing, 100035 China

**Keywords:** Cancer stem cells, Oncogenes, Mechanisms of disease

## Abstract

Recent studies have demonstrated that gastric cancer stem cells (CSCs) are a rare sub-group of gastric cancer (GC) cells and have an important role in promoting the tumor growth and progression of GC. In the present study, we demonstrated that the glycolytic enzyme Enolase 1 (ENO1) was involved in the regulation of the stem cell-like characteristics of GC cells, as compared to the parental cell lines PAMC-82 and SNU16, the expression of ENO1 in spheroids markedly increased. We then observed that ENO1 could enhance stem cell-like characteristics, including self-renewal capacity, cell invasion and migration, chemoresistance, and even the tumorigenicity of GC cells. ENO1 is known as an enzyme that is involved in glycolysis, but our results showed that ENO1 could markedly promote the glycolytic activity of cells. Furthermore, inhibiting glycolysis activity using 2-deoxy-d-glucose treatment significantly reduced the stemness of GC cells. Therefore, ENO1 could improve the stemness of CSCs by enhancing the cells’ glycolysis. Subsequently, to further confirm our results, we found that the inhibition of ENO1 using AP-III-a4 (ENOblock) could reduce the stemness of GC cells to a similar extent as the knockdown of ENO1 by shRNA. Finally, increased expression of ENO1 was related to poor prognosis in GC patients. Taken together, our results demonstrated that ENO1 is a significant biomarker associated with the stemness of GC cells.

## Introduction

Gastric cancer (GC) is the fifth most prevalent malignant neoplasm and the third most deadly carcinoma worldwide, based on WHO GLOBOCAN reporting^1^. An estimated one million new GC cases and nearly 600,000 deaths due to GC are diagnosed with each year^2,3^. The five-year survival rate of GC patients is <30%, because of tumor aggressiveness, metastasis, chemotherapy resistance, and relapse^4–6^. Cancer stem cells (CSCs) are characterized by their self-renewing ability and demonstrated pluripotent differentiation ability, which has been verified to contribute to cancer drug resistance, metastasis, and recurrence^7^. Numerous researchers have proved that CSCs are present in many types of tumors, such as breast cancer, brain tumors, and gastric cancer^8–12^. In 2009, Takaish et al.^12^ first isolated and identified gastric cancer stem cells (GCSCs) from gastric carcinoma cell lines. The source of GCSCs may be related to gastric epithelial cells^13^. With the characteristics of self-regeneration and pluripotent differentiation, GCSCs are associated with the occurrence and development of GC^14^. Furthermore, numerous signaling pathways and functions have been investigated, and results show that GCSCs are the primary causes of invasiveness, drug resistance, and metastasis in GC^15–17^.

Known as the Warburg effect, aerobic glycolysis is both a hallmark of cancer cells and the basis of various cancer cell’s biological characteristics^18^. In many types of tumors, the Warburg effect leads to a rise in total glycolysis not only in normal oxygen conditions but also in hypoxic conditions^18,19^. Thus, the Warburg effect may create a positive environment for cancer cells to divert nutrients^20^ for proliferation, metastasis, and drug resistance. Recent studies have demonstrated that glycolytic enzymes such as Enolases have a critical role in glycolysis in cancer cells^21^. ENO1, one of four types of Enolase isozymes, has been detected in almost all mature tissues^22,23^. The functions of ENO1 is now considered to be both a plasminogen receptor, which can promote inflammatory responses in several tumors^24^ and a glycolytic enzyme, which participates in catalyzing the penultimate step in glycolysis^23^. As a glycolysis enzyme, ENO1 can be overexpressed and activated by several glucose transporters and glycolytic enzymes that participate in the Warburg effect in cancer cells^25^. Moreover, ENO1 is thought to be related to aerobic glycolysis levels in tumor cells and malignant tumor development^26^. Recent studies have shown that ENO1 has a pivotal role in different tumor tissues, such as head and neck cancers, Non-Hodgkin’s lymphoma, breast cancer, cholangiocarcinoma, glioma, and GC^27–29^. For example, overexpression of ENO1 can promote tumor growth in hepatocellular carcinoma, and head and neck cancers, and functions as a potential oncogenic factor^29,30^. Furthermore, ENO1 was shown to influence proliferation, metastasis, and drug resistance in cancer cells by participating in the Warburg effect^31,32^. These studies indicated that ENO1 functioned as a potential oncogenic factor in endometrial carcinoma by inducing glycolysis^33^. It was also demonstrated that ENO1 was the center of a protein–protein interaction network composed of 74 GC-associated proteins and inhibition of ENO1 led to the growth inhibition of GCs^34^. Moreover, many studies have demonstrated that the overexpression of ENO1 contributes to the occurrence and development of GC. For example, ENO1 is related to the proliferation and metastasis of GCs^35^. In addition, overexpression of ENO1 can promote cisplatin resistance by enhancing glycolysis in GCs, while in contrast, inhibition of ENO1 can increase the sensitivity of GCs to chemotherapy by repressing glycolysis^36^.

Importantly, although ENO1 has been shown to be associated with the occurrence and progress of GC and take part in glycolysis in GCs, far less is known about the role of ENO1 in GCSCs. We, therefore, investigated the relationship between ENO1 and the stem cell-like characteristics of GC cells. We found that the expression of ENO1 was significantly increased in spheroids of GC cells. In addition, we discovered that ENO1 could promote the stemness of GC cells by enhancing glycolysis levels. Therefore, ENO1 was shown to be a possible biomarker of GCSCs, and targeting ENO1 could, therefore, be a valuable tool for improving the prognosis of GC patients.

## Materials and methods

### Cell culture and clinical samples

The human GC cell lines PAMC-82 and SNU16 were obtained from the Chinese Academy of Sciences. The PAMC-82 cell line was cultured in Dulbecco’s modified Eagle’s medium (DMEM, Invitrogen, Carlsbad, CA, USA) containing 10% fetal bovine serum. The SNU16 cell line was cultured in RPMI-1640 medium. All of the cell lines were confirmed to be free of mycoplasma contamination after testing with the kit from Shanghai Yise Medical Technology (MD001). The commercial tissue microarrays were constructed by Shanghai Biochip Co. Ltd. The study was approved by the medical ethics committee of Cancer Hospital, Chinese Academy of Medical Sciences (Beijing, China) (Ethical approval number: NCC1999 G-003).

### Self-renewal assay

We used these spheroid-formation experiments to explore the self-renewal capacity of these cells. The cells were seeded in 24-well ultra-low attachment plates (Corning) at a density of 500 cells/well and cultured in SFM that was supplemented with 0.8% methylcellulose (Sigma), 20 ng/mL EGF, B27 (1:50), 10 ng/mL LIF, and 20 ng/mL bFGF. The cells were cultured at 37 °C in 5% CO_2_ for 7–13 days, and then the quantity of spheroids was counted using a microscope.

### Antibodies for western blot and immunohistochemistry

The ENO1 antibody (ab227978), β-Tubulin antibody (ab52901), anti-CD44 antibody (ab157107), SOX2 antibody (ab97959), anti-Nanog antibody (ab80892), and anti-Oct4 antibody (ab18976) were from Abcam.

### Transwell™ invasion assay

To evaluate the invasive activity, a total of 2 × 10^4^ serum-starved cells were resuspended in 200 μL SFM and plated in the top of a Transwell™ chamber (24-well insert; pore size, 8 μm; Corning) that was coated with diluted Matrigel (BD Biosciences). After 24 h, the number of infiltrating cells was counted using a light microscope, and the invasion of cells was analyzed quantitatively.

### Chemosensitivity assay

Cells were seeded in 96-well plates (4000 cells/well) and cultured for 24 h. Then the cells were treated with different concentrations of cisplatin for 72 h. A Cell Counting Kit-8 (CCK8) was used to evaluate the number of viable cells, and the absorbance at 450 nm was measured using a microplate reader (Bio-rad, USA).

### Tumorigenicity in BALB/c nude mice

BALB/c nude mice (4–5 weeks old) were obtained from HFK Bioscience Company (Beijing, China). For tumorigenesis assays, 2.4 × 10^6^ cells were subcutaneously injected into the back of nude mice (5 mice/group). The tumor size was recorded every week. All mice were then sacrificed on day 30 after inoculation and the tumor weight of each mouse was measured.

### Glucose consumption

We seeded cells in six-well plates for 24 h and then replaced the medium with 3 mL of fresh medium. After a fixed time, we collected the supernatant and measured glucose consumption using a Glucose and Sucrose Assay Kit (Sigma-Aldrich, MAK013). The number of cells was counted three times. The glucose consumption was normalized to μmol/10^6^ cells.

### Lactic acid measurement

Cells were collected after culturing for the same length of time as indicated above, and then lactic acid production of cells was measured by colorimetry according to the instructions of a Lacate Colormetric Assay Kit II (Biovision, K627-100). The cells left were counted and the lactic acid production was normalized to μmol/10^6^ cells.

### Glycolysis level analysis

Cells were plated in a Seahorse XF96 plate at a density of 15,000 cells per well, and the compounds that included glucose, oligomycin, and 2-deoxy-d-glucose (2-DG) were loaded into appropriate ports of a hydrated sensor cartridge. Finally, the cells’ glycolysis stress was tested using the Seahorse XFe/XF Analyzer.

### Statistical analysis

All data were shown as the mean ± standard deviation (SD) derived from at least three independent experiments. The statistical significance was calculated by unpaired Student’s *t*-tests and results were considered significant if *P* < 0.05. SPSS 13.0 and GraphPad Prism 5.0 were used to perform all analyses.

## Results

### ENO1 is related to the stemness of GCs

**Fig. 1:**
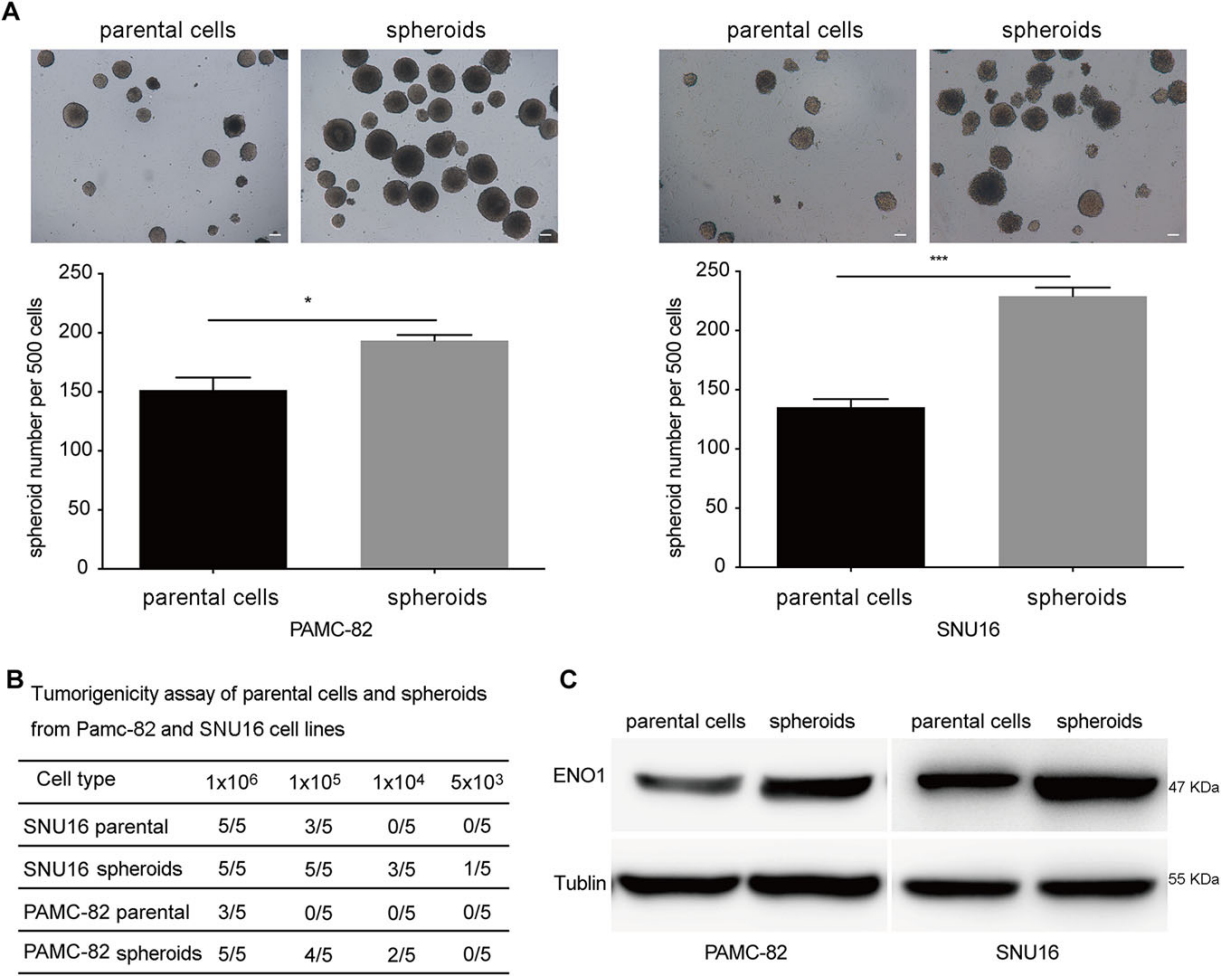
Enolase 1 (ENO1) is upregulated in stem cell-like cells enriched from PAMC-82 and SNU16 cells by spheroid-forming culture. **A** Analysis of the self-renewal abilities of PAMC-82, SNU16 parental cells, and spheroids using methylcellulose spheroid-formation assay. Scale bar, 100 μm. **B** Tumorigenicity assay in PAMC-82, SNU16 parental cells, and spheroids. **C** Western blot analysis for the expression of ENO1 in PAMC-82, SNU16 parental cells, and spheroids. spheroids: GSCSs enriched from parental cells cultured in spheroid-formation conditions and passaged to the third-passage. Results are from representative experiments in triplicate and shown as the mean ± standard deviation (SD). *P < 0.05, **P < 0.01, ***P < 0.001.

Considering that CSCs only account for a small fraction of the heterogeneous GC cell lines PAMC-82 and SNUU16, we enriched GCSCs by performing a spheroid-forming culture of both PAMC-82 and SNU16 cells. After 7–10 days, both cell lines could form non-adherent spheres containing between 40 and 100 cells, we called them “spheroids”. These spheroids could be continuously passaged, and third-passage spherical cells were used in all relevant experiments. To determine if spheroids can be considered as CSCs, we measured the important characteristics of CSCs in spheroids compared with parental cells. A self-renewal assay showed that the capacity of self-renewal in spheroids was superior to parental cells, as the spheroids had markedly increased the number of colonies when compared to parental cells (Fig. 1A). Then, we assessed the tumorigenicity of these spheroids and parental cells (PAMC-82 and SNU16) using a xenograft model. The results indicated that the same number of spheroid cells could possess a stronger tumorigenic ability as compared to parental cells, thus spheroids had a higher tumorigenic potential (Fig. 1B). Taken together, these results demonstrated that spheroids are CSC-like cells. To verify whether ENO1 could be related to the stemness of GC cells, we investigated the ENO1 expression in spheroids as compared to parental cells (PAMC-82 and SNU16) using western blotting. Results demonstrated that the expression of ENO1 in spheroids was significantly higher than that in parental cells (Fig. 1C). In a word, these findings indicated that ENO1 could be related to the stem cell-like properties of GC cells.

### ENO1 promotes the stem-like characteristics of GCs

**Fig. 2:**
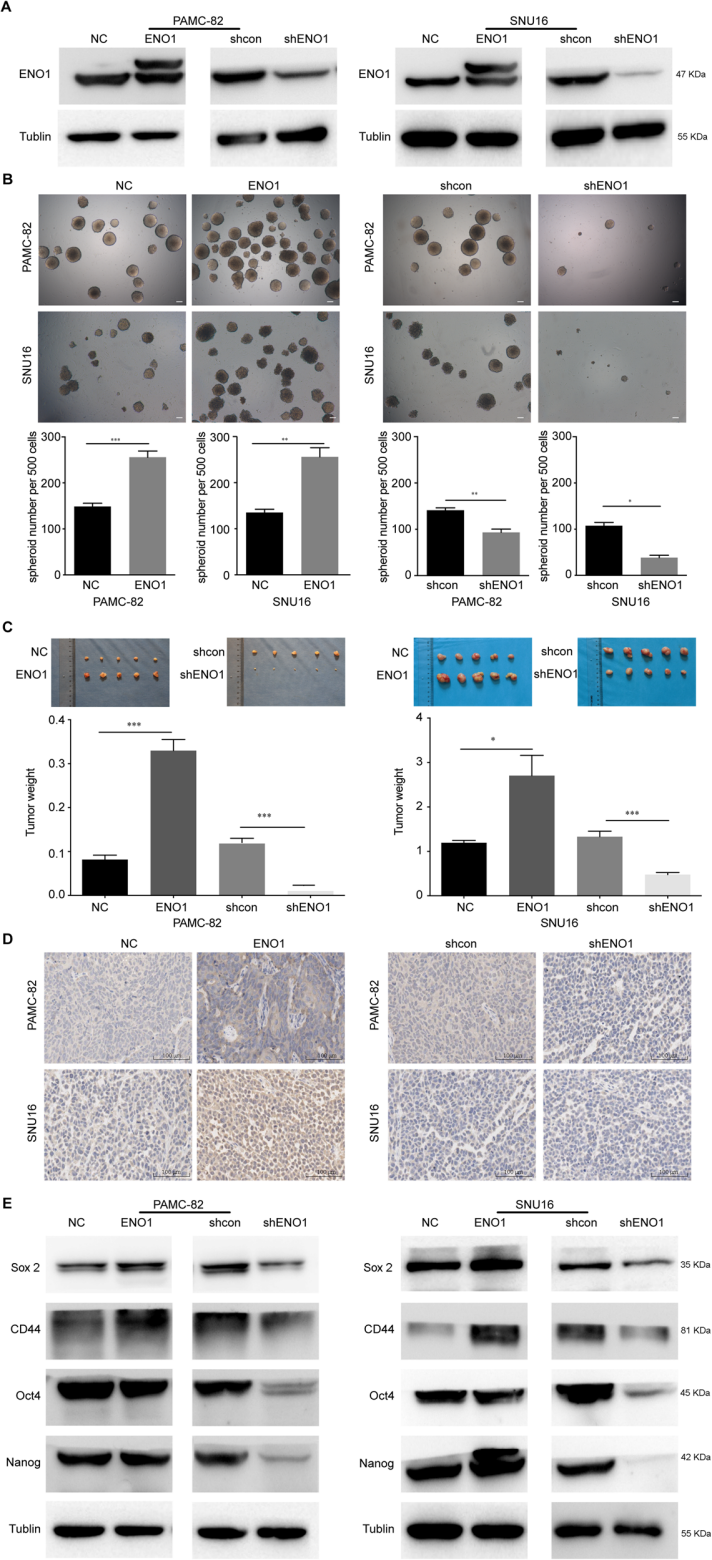
Enolase 1 (ENO1) promotes the stem-like characteristics of gastric cancers (GCs). **A** Western blot analysis of the expression of ENO1 in PAMC-82 and SNU16 cells stably expressing pLenti-NC, pLenti-ENO1, shcon, or shENO1. **B** Analysis of the self-renewal abilities of PAMC-82 and SNU16 cells stably expressing pLenti-NC, pLenti-ENO1, shcon, or shENO1. Scale bar, 100 μm. **C** Tumorigenicity of PAMC-82 and SNU16 cells stably expressing pLenti-NC, pLenti-ENO1, shcon, or shENO1. **D** IHC for ENO1 in serial sections of tumor tissues from mice injected with PAMC-82 or SNU16 cells stably expressing pLenti-NC, pLenti-ENO1, shcon, or shENO1. Scale bar, 100 μm. **E** Expression of stem cell markers in PAMC-82 and SNU16 stable cell lines was detected by western blot. Results are from representative experiments in triplicate and shown as the mean ± standard deviation (SD). *P < 0.05, **P < 0.01, ***P < 0.001.

To explore the impact of ENO1 on stem-like characteristics, we first used retroviral transduction technology to stably knockdown and overexpress ENO1 in PAMC-82 and SNU16 cells, and confirmed these perturbations by the Western blot (Fig. 2A). Next, we used these stable cell lines to determine the role of ENO1 in stem cell-like characteristics. First, we investigated the impact of ENO1 on the capacity of self-renewal. Upon ENO1 overexpression (pLenti-ENO1), the ability of self-renewal in PAMC-82 and SNU16 cells were significantly increased (Fig. 2B). In contrast, the capacity for self-renewal in ENO1 knockdown (shENO1) cells was markedly decreased as compared to control cells (Fig. 2B). In order to expand our observations in vitro, we explored whether ENO1 could affect the tumorigenicity of GC cells in vivo. pLenti-ENO1, shENO1, and corresponding control cells (pLenti-NC, shcon) were injected subcutaneously into nude mice. We found that tumors derived from the pLenti-ENO1 cells grew faster and weighed more and the tumors derived from shENO1 cells grew slower and were much smaller in weight compared with those originating from corresponding control cells (Fig. 2C). In addition, IHC experiments confirmed the pattern of ENO1 expression in the above-mentioned tumors (Fig. 2D). These results demonstrated that pLenti-ENO1 cells possessed much stronger tumorigenic potentials and shENO1 cells possessed much weaker tumorigenic potentials. Moreover, we found that the expression of the stem cell markers CD44, Nanog, Oct4, and Sox2 increased in pLenti-ENO1 cells while these markers all decreased in shENO1 cells (Fig. 2E). Taken together, these results suggested that ENO1 could enhance the CSC-like characteristics of GC cells.

### ENO1 promotes characteristics associated with stemness in GCs

**Fig. 3:**
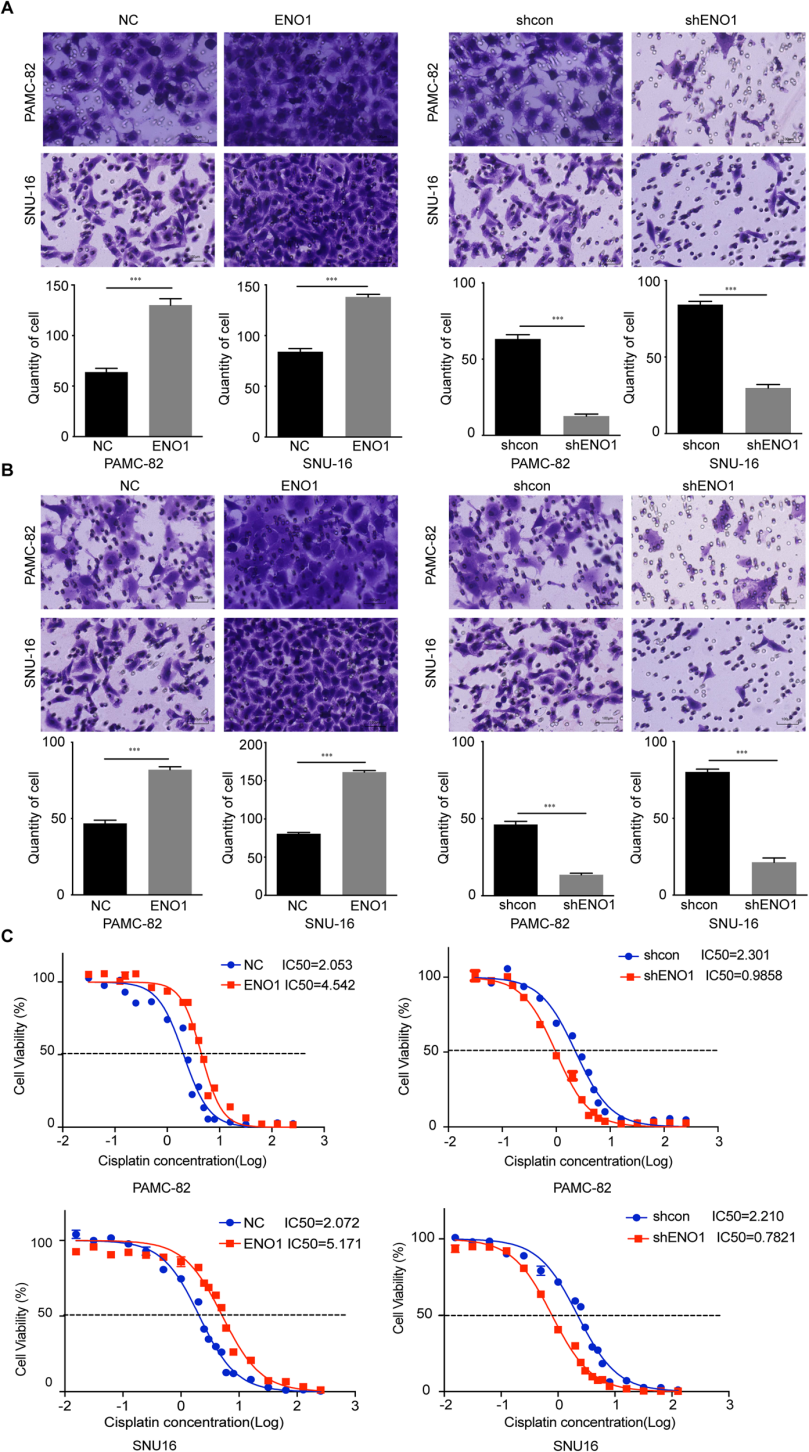
Enolase 1 (ENO1) promotes characteristics associated with stemness in gastric cancers (GCs). **A** Matrigel invasion assay of PAMC-82 and SNU16 cells stably expressing pLenti-NC, pLenti-ENO1, shcon, or shENO1. Scale bar, 100 μm. **B** Migration assay of PAMC-82 and SNU16 cells stably expressing pLenti-NC, pLenti-ENO1, shcon, or shENO1. Scale bar, 100 μm. **C** PAMC-82 and SNU16 stable cells were treated with several different concentrations of cisplatin (0.015625–256 μM) for 72 h. The cell viability was determined by CCK8. Results are from representative experiments in triplicate and shown as the mean ± standard deviation (SD). *P < 0.05, **P < 0.01, ***P < 0.001.

Several studies have demonstrated that high metastasis and drug resistance may be important characteristics of stem-like cancer cells. Thus, we performed Transwell™ assays to determine the invasion and migration potentials of pLenti-ENO1 and shENO1 cells. Compared with the control group (pLenti-NC, and shcon), the migration and invasion rates of pLenti-ENO1 cells were higher, while those of shENO1 cells were significantly lower (Fig. 3A, B). We then determined the effect of ENO1 on cisplatin resistance. Our results indicated that the overexpression of ENO1 significantly decreased the cisplatin sensitivities of PAMC-82 and SNU16 cells (Fig. 3C). Inversely, knockdown of ENO1 resulted in a marked increase of cisplatin sensitivity (Fig. 3C).

### ENO1 increases the stemness of GC cells through the promotion of glycolysis

**Fig. 4:**
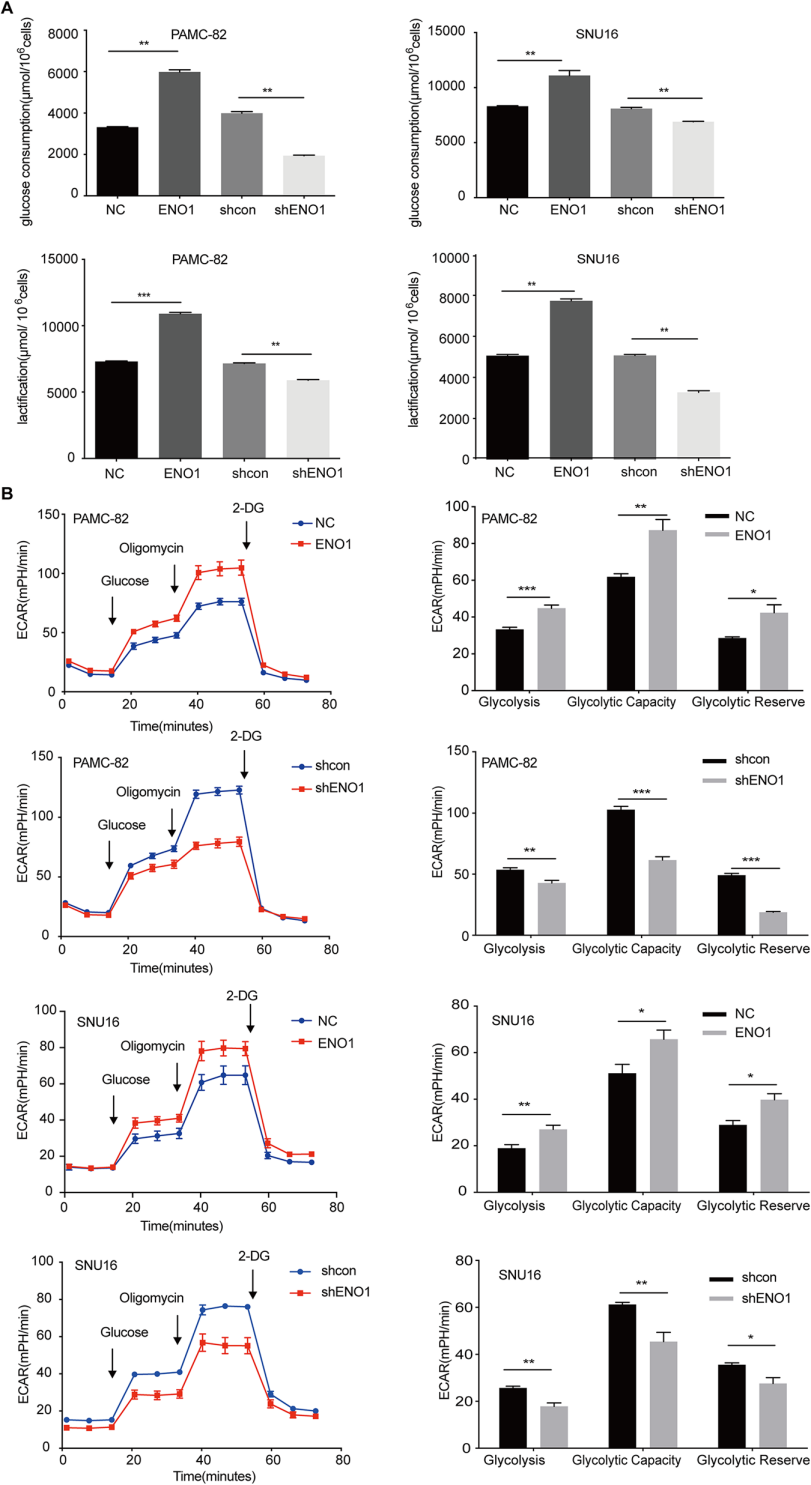
Enolase 1 (ENO1) increases the stemness of gastric cancer (GC) cells via glycolysis promotion. **A** PAMC-82 and SNU16 cells stably expressing pLenti-NC, pLenti-ENO1, shcon, or shENO1 were cultured for 36 h, then the levels of glucose consumption and lactic acid production were measured according to the cell numbers. Fold changes were normalized (μmol/10^6^ cells). **B** Extracellular acid ratio (ECAR) was measured by Seahorse XF in PAMC-82 and SNU16 cells stably expressing pLenti-NC, pLenti-ENO1, shcon, or shENO1. ECAR curves from cells treated with glucose, oligomycin, and 2-DG. Black arrows indicate the time point of cell treatment. Results are from representative experiments in triplicate and shown as the mean ± standard deviation (SD). *P < 0.05, **P < 0.01, ***P < 0.001.

As it is well-known that ENO1 is an important enzyme for catalyzing 2-phosphoglycerate to phosphoenolpyruvate in the glycolysis pathway, we wondered whether ENO1 could affect the stemness of cells by enhancing glycolysis. We explored the changes to glycolysis in overexpression and knockdown cells compared with their corresponding control cells. The results showed that glucose consumption and lactic acid production were increased in ENO1 overexpression cells (Fig. 4A). On the contrary, after stably silencing ENO1, glucose consumption, and lactic acid production were both markedly decreased (Fig. 4A). To further confirm that ENO1 could influence glycolytic metabolism, we determined the extracellular acidification rate (ECAR) of these stable cell lines. Consistent with our hypothesis, overexpression of ENO1 increased the ECAR levels (Fig. 4B). Meanwhile, decreased ECAR levels were observed in ENO1 knockdown cells (Fig. 4B).

### Glycolysis level significantly related with the stemness of GCs

**Fig. 5:**
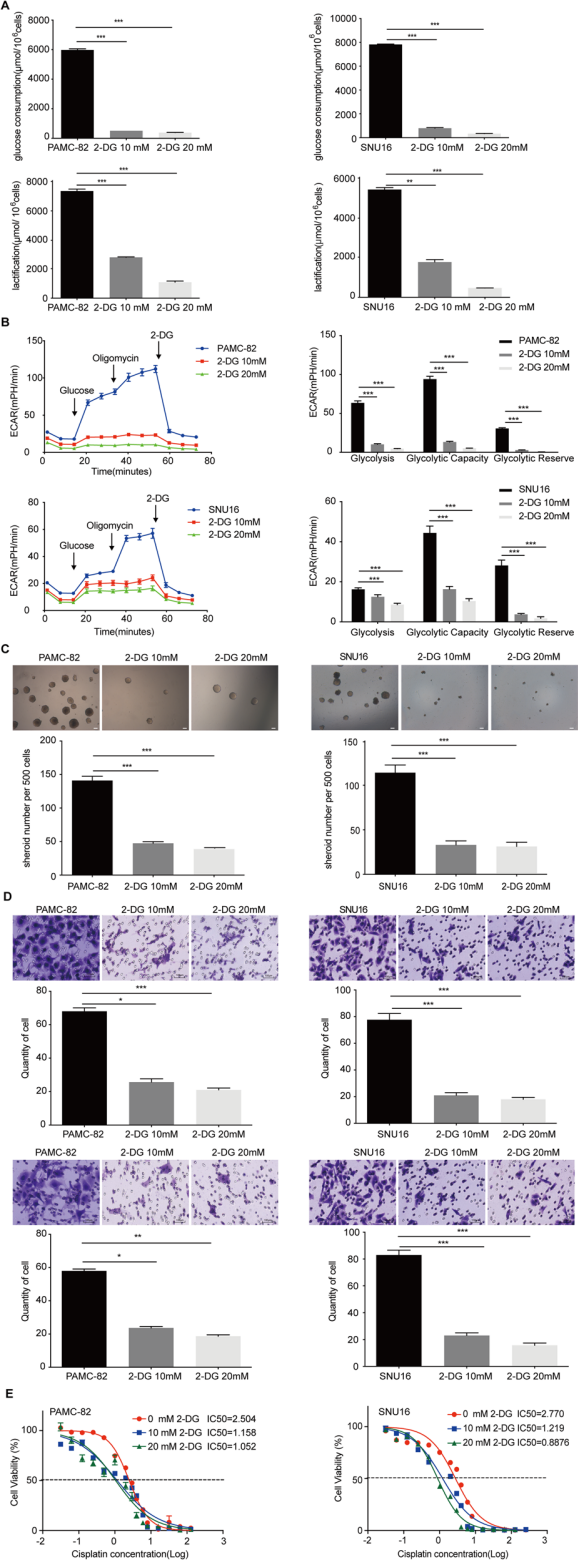
Treatment with 2-DG inhibits glycolysis and stem cell-like characteristics in gastric cancers (GCs). **A** PAMC-82 and SNU16 cells were cultured in 10 or 20 mM 2-DG for 36 h, and the levels of glucose consumption and lactic acid production were measured according to the cell numbers. Fold changes were then normalized (μmol/10^6^ cells). **B** Extracellular acid ratio (ECAR) of cells was measured by Seahorse XF in PAMC-82 and SNU16 cells treated with 10 or 20 mM 2-DG for 36 h. ECAR curves of cells treated with glucose, oligomycin, or 2-DG. Black arrows indicate the time point of cell treatment. **C** Analysis of the self-renewal abilities of PAMC-82 and SNU16 cells cultured in 10 or 20 mM 2-DG for 24 h. Scale bar, 100 μm. **D** The migration and invasion assay of PAMC-82 and SNU16 cells treated with 10 or 20 mM 2-DG for 24 h (above: migration, below: invasion). Scale bar, 100 μm. **E** PAMC-82 and SNU16 cells cultured in 10 or 20 mM 2-DG for 24 h, in the presence of several different concentrations of cisplatin (0.015625–256 μM) for 72 h. Cell viability was measured by CCK8. Results are from representative experiments in triplicate and shown as the mean ± standard deviation (SD). *P < 0.05, **P < 0.01, ***P < 0.001.

To determine whether the glycolysis level could affect the CSC-like characteristics of GC cells, we treated PAMC-82 and SNU16 cells with the glycolytic inhibitor 2-DG and confirmed whether the glycolysis level could be inhibited in these cells. Our results demonstrated that treatment with 2-DG (10 or 20 mM) could markedly inhibit the glycolysis level since glucose consumption and the production of lactic acid were decreased by 2-DG treatment (Fig. 5A). Moreover, we found that 2-DG treatment (10 or 20 mM) could significantly decrease the ECAR levels of these cells (Fig. 5B). We then studied the stem cell-like characteristics of cells that were treated with 2-DG as compared with corresponding basal cells. Firstly, our results demonstrated that 2-DG treatment at 10 or 20 mM could observably decrease the self-renewal capacity of both cells (Fig. 5C). Then we tested the function of 2-DG on cell migration and invasion behaviors and found that 2-DG treatment at 10 or 20 mM all markedly inhibited migration and invasion rates (Fig. 5D). Finally, this indicated that treatment with 2-DG (10 or 20 mM) could strongly increase the cisplatin sensitivity in PAMC-82 and SNU16 cells (Fig. 5E). Furthermore, these studies suggested that the inhibition of glycolysis was related to the stemness of GCs.

Taken together, these results demonstrated that overexpression of ENO1 could enhance glycolysis to promote the stemness of cells while knockdown of ENO1 could inhibit glycolysis to reduce the stemness of cells. Thus, ENO1 can regulate glycolysis levels to influence the stem cell-like characteristics of GCs.

### ENO1 inhibitor (ENOblock) inhibits the stemness of GC cells

**Fig. 6:**
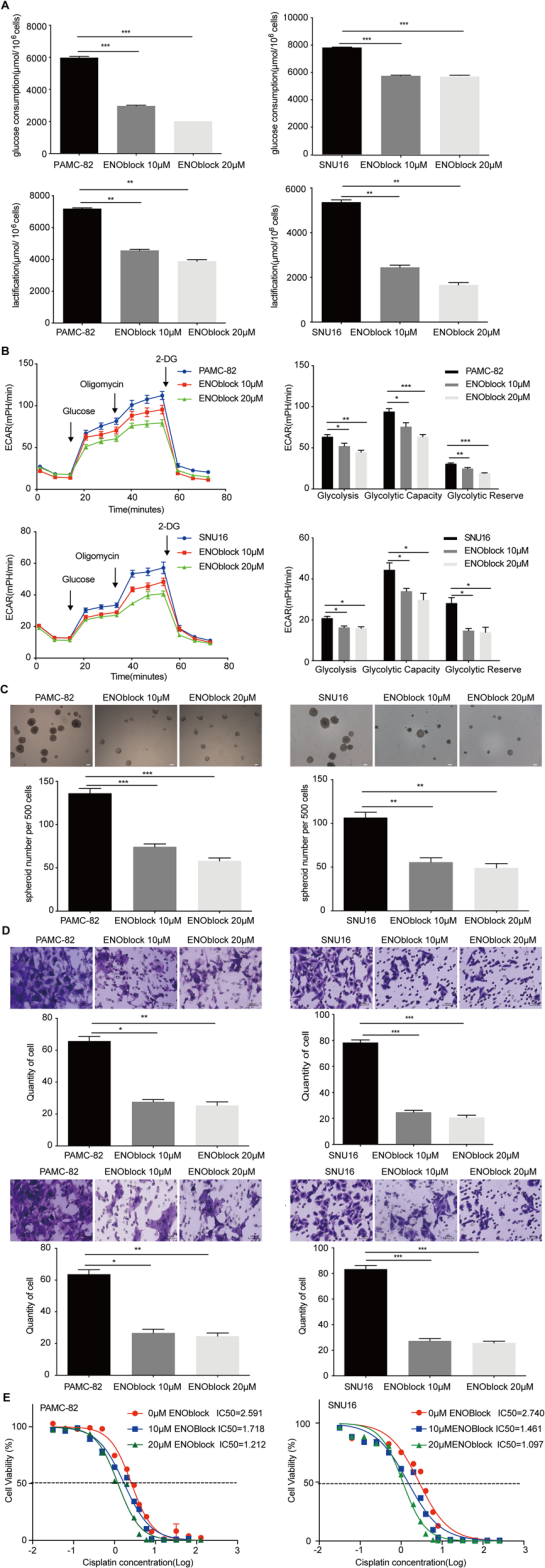
Treatment with ENOblock inhibits the stemness of gastric cancers (GCs). **A** PAMC-82 and SNU16 cells were cultured in 10 μM or 20 μM ENOblock for 36 h, and the levels of glucose consumption and lactic acid production were measured according to the cell numbers. Fold changes were then normalized (μmol/10^6^ cells). **B** Extracellular acid ratio (ECAR) of cells was measured by Seahorse XF in PAMC-82 and SNU16 cells treated with 10 or 20 μM ENOblock for 36 h. ECAR curves of cells treated with glucose, oligomycin, or 2-DG. Black arrows indicate the time point of cell treatment. **C** Analysis of self-renewal in PAMC-82 and SNU16 cells cultured in 10 or 20 μM ENOblock for 24 h. Scale bar, 100 μm. **D** Migration and invasion abilities of PAMC-82 and SNU16 cells treated with 10 or 20 μM ENOblock for 24 h (above: migration, below: invasion). Scale bar, 100 μm. **E** PAMC-82 and SNU16 cells cultured in 10 or 20 μM ENOblock for 24 h, in addition to several different concentrations of cisplatin (0.015625–256 μM) for 72 h before harvest. The cell viability was measured by CCK8. Results are from representative experiments in triplicate and shown as the mean ± standard deviation (SD). *P < 0.05, **P < 0.01, ***P < 0.001.

AP-III-a4 (ENOblock) is a well-known inhibitor of ENO1. To extend our observations as before, we used ENOblock to inhibit the activity of ENO1 in PAMC-82 and SNU16 cells and then investigated the changes of stemness in GCs. We found that treatment with ENOblock (10 or 20 μM) could reduce the glycolysis level, that is, ENOblock could decrease glucose consumption and lactic acid production (Fig. 6A). Moreover, ENOblock treatment (10 or 20 μM) significantly decreased the ECAR levels of these cells (Fig. 6B). Furthermore, ENOblock treatment at 10 or 20 μM could significantly inhibit GCs’ self-renewal capacity (Fig. 6C). We then explored the function of ENOblock on cell migration and invasion. Our results indicated that treatment with ENOblock (10 or 20 μM) could strongly reduce cells’ migration and invasion rates (Fig. 6D). Moreover, cells’ cisplatin sensitivities were markedly increased by treatment with ENOblock at 10 or 20 μM (Fig. 6E). These results suggested that the function of inhibition by ENOblock was in consistent with the function of ENO1-knockdown.

### ENO1 is a predictor of poor prognosis in clinical cases of GC

**Fig. 7:**
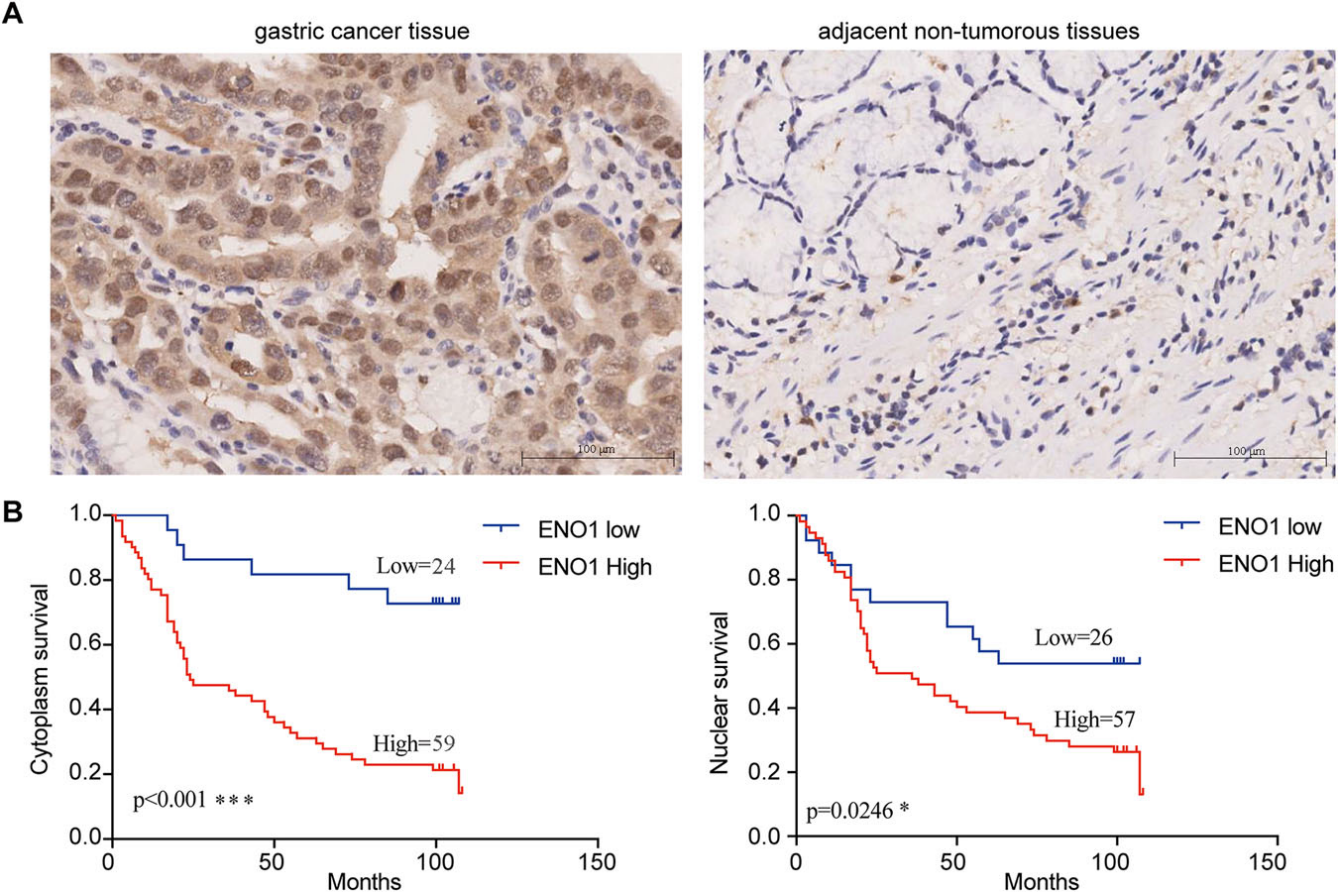
Enolase 1 (ENO1) is a predictor of poor prognosis in clinical cases of gastric cancer (GC). **A** Expressions of ENO1 in normal and GC cancer tissues were detected by IHC. Scale bar, 100 μm. **B** Overall survival of GC patients with negative or positive ENO1 expression. *P < 0.05, **P < 0.01, ***P < 0.001.

To explore the clinical significance of ENO1 in the development of GC, we determined the expression of ENO1 in GCs and their adjacent non-tumorous tissues by IHC. Our results showed that ENO1 expression was positive in 59/83 primary tumors (71.1%), but weak or nonexistent in adjacent normal tissues (Fig. 7A). Table 1 summarizes the relationship between ENO1 expression level and clinicopathological characteristics in patients with GC. Interestingly, our analysis demonstrated that high levels of endochylema ENO1 were markedly correlated with infiltration depth (*P* = 0.038). We also found that the levels of nuclear ENO1 expression were markedly correlated with Stage (*P* = 0.023). Nevertheless, there were no statistically significant correlations between ENO1 expression and other clinicopathologic features (Table 1). Kaplan–Meier analysis was used to test whether ENO1 expression was related to the survival of GC patients. This analysis indicated that the overall survival of patients with GC with high levels of ENO1 in the cytoplasm and nucleus were all significantly shorter than those with low or no ENO1 expression (Fig. 7B). In summary, these observations showed that the levels of ENO1 might have an important role in GC progression.

**Table 1:** Correlation of ENO1 expression in GC tissues with clinicopathological parameters.

Characteristics	Case (*N*)	ENO1 (endochylema)			ENO1 (nuclear)		
		Positive *N* (%)	*χ*^2^	*P*-value	Positive *N* (%)	*χ*^2^	*P*-value
Age			1.039	0.308		0.701	0.403
<60	31	20 (64.5)			23 (74.2)		
≥60	52	39 (75.0)			34 (65.4)		
Gender			0.315	0.575		0.378	0.538
Male	55	38 (69.1)			39 (70.9)		
Female	28	21 (75.0)			18 (64.3)		
Clinical stages			1.373	0.241		5.140	0.023
1 + 2	30	19 (63.3)			16 (53.3)		
3 + 4	53	40 (75.5)			41 (77.4)		
Depth of invasion			4.287	0.038		0.351	0.553
T1 + T2	16	8 (50.0)			10 (62.5)		
T3 + T4	67	51 (76.1)			47 (70.1)		
Lymph node involvement			0.755	0.385		3.737	0.53
N0 + N1	33	21 (63.6)			18 (54.5)		
N2 + N3	50	38 (76.0)			39 (78.0)		
Metastasis			1.557	0.212		0.19	0.891
M0	74	51 (68.9)			51 (68.9)		
M1	9	8 (88.9)			6 (66.7)		
Pathological grading			0.712	0.399		0.38	0.845
I + II	53	36 (67.9)			36 (67.9)		
III + IV	30	23 (76.7)			21 (70.0)		

*ENO1* Enolase 1, *GC* gastric cancer.

## Discussion

In recent years, an increasing number of reports have confirmed the existence and importance of CSCs in GC^37,38^. As we all know, CSCs are a small population of tumor cells, which are characterized by self-renewal capacity, higher tumorigenicity, multiple differentiation, and drug resistance^39–41^. Stem cell markers are also overexpressed in CSCs such as CD44, Oct4, Lgr5, CD24, and CD133^12^. These cells are linked with tumor hierarchy, initiation, heterogeneity, and propagation^38^. Spherical cell culture is a mature stem cell-like cell formation technique^9^. CSCs in GC tissues and cell lines have been sorted successfully using this method^39^.

In this study, we obtained GCSCs (spheroids) from the GC cell lines PAMC-82 and SNU16, and we found that these spheroids were characterized by the enhanced capacity of self-renewal and tumorigenicity compared with their respective parental cell lines. Interestingly, we found that ENO1 upregulated in spheroids compared with parental cells, suggesting that ENO1 was possibly associated with these cells’ stem-like characteristics.

Enolases have three isoenzyme forms, namely alpha-enolase, beta-enolase, and gamma-enolase^42^. Alpha-enolase (ENO1) is mainly present in almost all adult tissues. ENO1 is not only an important enzyme in the glycolysis pathway, catalyzing the dehydration of 2-phosphate-d-glycerate to form phosphoenolpyruvate, but also a plasminogen receptor on the surface of various cells^43,44^. However, in this study, we only focused its enzymatic role and function. Recently, It has been shown that ENO1 expression is abnormal in many human cancers, including glioma, colorectal cancer, pancreatic cancer, lung cancer, and head and neck cancers^28,29,31,45,46^. Furthermore, previous studies have demonstrated that ENO1 was overexpressed in GC tissues and was related to the progression and prognosis of GC^35,36^. In this study, we further demonstrated that ENO1 expression was significantly associated with the overall survival of GC patients, implying the important functions of ENO1 in GC progression.

Studies focusing on the relationship of ENO1 to CSCs are scarce, including GCSCs. In the present study, we addressed whether ENO1 was associated with GC cells’ stem cell-like characteristics. We found that overexpression of ENO1 could increase GC cells’ stem cell-like characteristics, including their self-renewal capacity, migration and invasion rates, tumorigenicity, and drug resistance. Moreover, the levels of stem cell markers were enhanced in these cells, such as CD44, OCT4, Sox2, and Nanog. On the contrary, the silencing of ENO1 by shRNA could inhibit GC cells’ stemness and decreased the levels of these markers. Furthermore, we confirmed these results using the ENO1 inhibitor ENOblock. These results indicated that inhibition of ENO1 by ENOblock also could inhibit the stem-like characteristics of GC cells to a similar agree as the silencing of ENO1 by shRNA. Taken together, ENO1 could markedly regulate GC cells’ stemness.

ENO1 is considered to be an important enzyme in the glycolytic pathway, but it is not the rate-determining enzyme in glycolysis. To further evaluate the effect of ENO1 on the glycolysis pathway in GC cells, we analyzed the glycolysis changes caused by ENO1. The results of our analysis of glucose consumption and lactic acid production of stable GC cells showed that overexpression of ENO1 significantly enhanced cells’ capability for glycolysis. We also demonstrated that the silencing of ENO1 decreased the glycolysis capacity of GC cells. These results showed that ENO1 could increase the stemness of GC cells by enhancing the glycolysis capacity of cells.

The phenomenon of increased glycolysis rate in tumor cells is called the Weinberg effect^47^. The significance of glycolysis has been increasingly demonstrated in many diverse cancers, including GC. Recent studies have revealed that increased glycolysis levels possibly contribute to the development of cancer cells^48–52^. For example, ENO1 enhances the level of glycolysis to promote GC cells’ resistance to chemotherapy^27^. Moreover, accelerated glycolysis increases the proliferation and invasion of non-small cell lung cancer^31^. Previous studies have demonstrated that the enhancement of aerobic glycolysis markedly promotes cancer cell growth and development^32,53,54^. However, studies focused on the association of glycolysis levels and stem-like characteristics of GC cells are scarce to nonexistent. In this study, we inhibited GC cells’ glycolysis capacity by using 2-DG, and then explored the changes of stemness in GC cells. Our analysis of glucose consumption and lactic acid production confirmed that treatment with 2-DG significantly inhibited the glycolysis of GC cells. We also found that inhibiting glycolysis could decrease their capacity for self-renewal, invasion, and resistance to chemotherapy. In summary, inhibition of glycolysis could markedly reduce the stemness of GC cells. Taken together, these results indicated that ENO1 could increase the stemness of GC cells by enhancing the glycolysis capacity of cells.

In conclusion, our study illustrated that ENO1 was upregulated in GC spheroid cells that were characterized by increased stemness compared with parental cells, and its upregulation was associated with poor prognosis in GC patients. Functionally, ENO1 could promote the stem-like characteristics of GC cells by prominently regulating tumor glycolysis. Our data demonstrated that ENO1 was connected with the stemness of GC cells and could be used as a predictive biomarker for GCSCs. Future work should illustrate if it is possible to use ENO1 for prognosis and as a therapeutic target in GC.
